# Pretreatment Plasma EBV-DNA Load Guides Induction Chemotherapy Followed by Concurrent Chemoradiotherapy in Locoregionally Advanced Nasopharyngeal Cancer: A Meta-Analysis

**DOI:** 10.3389/fonc.2020.610787

**Published:** 2021-02-16

**Authors:** Lin Lai, Xinyu Chen, Chuxiao Zhang, Xishan Chen, Li Chen, Ge Tian, Xiaodong Zhu

**Affiliations:** ^1^ Department of Radiotherapy, Guangxi Medical University Cancer Hospital, Nanning, China; ^2^ Department of Medical Oncology, Ruikang Hospital Affiliated to Guangxi University of Chinese Medical, Nanning, China; ^3^ Department of Medical Oncology, The First Affiliated Hospital of Guangxi Medical University, Nanning, China; ^4^ Department of Oncology, Wuming Hospital of Guangxi Medical University, Nanning, China

**Keywords:** pretreatment plasma EBV-DNA load, locoregionally advanced nasopharyngeal cancer, induction chemotherapy, concurrent chemoradiotherapy, meta-analysis

## Abstract

**Background:**

The efficacy of induction chemotherapy (IC) followed by concurrent chemoradiotherapy (CCRT) in locoregionally advanced nasopharyngeal cancer (LA-NPC) is controversial. In this paper, we conduct a meta-analysis based on relevant studies to provide strong evidence for clinical strategies.

**Materials and Methods:**

We searched the MEDLINE, Embase, Cochrane, PubMed, and Web of Science databases for studies that stratified patients based on a high or low plasma Epstein–Barr virus deoxyribonucleic acid (EBV-DNA) load before treatment and compared the clinical efficacy of IC+CCRT vs. CCRT alone in LA-NPC. We tested for heterogeneity of studies and conducted sensitivity analysis. Subgroup analysis was performed for overall survival (OS), progression-free survival (PFS), distant metastasis-free survival (DMFS), and locoregional relapse-free survival (LRFS).

**Results:**

Seven studies with a total of 5289 cases were finally included in the meta-analysis. The heterogeneity test revealed the homogeneity of OS (*I* ^2 ^= 0.0%, *p*=0.794), PFS (*I* ^2 ^= 0.0%, *p*=0.778), DMFS (*I* ^2 ^= 0.0%, *p*=0.997), and LRFS (*I* ^2 ^= 0.0%, *p*=0.697) in patients with EBV-DNA loads of ≥4000 copies/ml in both the IC+CCRT and CCRT groups. The results reveal that IC+CCRT significantly extended the OS (HR 0.70 [95% CI 0.58-0.83], *p*=0.000), PFS (HR 0.83 [95% CI 0.70-0.99], *p*=0.033), and DMFS (HR 0.79 [95% CI 0.69-0.9], *p*=0.000) of patients compared with the CCRT group, but there were no beneficial effects on LRFS (HR 1.07 [95% CI 0.80-1.42], *p*=0.647). The heterogeneity test found that there was no significant heterogeneity of PFS (*I* ^2 ^= 0.0%, *p*=0.564), DMFS (*I* ^2 ^= 0.0%, *p*=0.648), LRFS (*I* ^2 ^= 22.3%, *p*=0.257), and OS (*I* ^2 ^= 44.6%, *p*=0.164) in patients with EBV-DNA loads of <4000 copies/ml. The results show that IC+CCRT prolonged DMFS (HR 0.57 [95% CI 0.39-0.85], *p*=0.006) of patients without significant improvements in OS (HR 0.88 [95% CI 0.55-1.26], *p*=0.240), PFS (HR 0.98 [95% CI 0.74-1.31], *p*=0.908), and LRFS (HR 0.98 [95% CI 0.54-1.77], *p*=0.943).

**Conclusions:**

Pretreatment plasma EBV-DNA can be considered a promising effective marker for the use of IC in LA-NPC patients. The addition of IC could improve the OS and PFS of patients with EBV-DNA load ≥4000 copies/ml, but we saw no efficacy in patients with EBV-DNA load <4000 copies/ml. Moreover, regardless of the EBV-DNA load, IC could improve DMFS, but there was no effect on LRFS.

## Introduction

Nasopharyngeal cancer (NPC) is an epithelial malignancy featuring a regional incidence as evidenced by the prevalence in Guangdong and Guangxi provinces in South China, Southeast Asia, and North Africa (1, 2). Non-keratinizing NPC is the main pathological type in high-incidence areas, and almost all patients have been infected with Epstein–Barr virus (EBV). Studies demonstrate that EBV is involved in the development of this malignancy and is considered a key pathogenic factor (3, 4). Therefore, EBV-related molecules are ideal biomarker candidates for NPC, of which plasma EBV-deoxyribonucleic acid (DNA) possesses high specificity and sensitivity and exhibits prominent perspectives in screening, diagnosis, and prognostic prediction of the disease (5).

According to the 7th or 8th edition of American Joint Committee on Cancer (AJCC), stage III–IVB NPC (AJCC 7th) or stage III–IVA NPC (AJCC 8th) is the most common advanced type with a high proportion of 70%–90% in newly diagnosed cases (6, 7). Because of its high radiosensitivity, intensity modulated radiation therapy (IMRT)-based concurrent chemoradiotherapy is confirmed as the main treatment for locoregionally advanced NPC (LA-NPC), providing 3- and 5-year overall survival (OS) rates of 76% and 72.3%, respectively (8–10). However, about 20%–30% of patients still develop relapses and metastases after standard treatment (11). Instead, concurrent chemoradiotherapy (CCRT)-based intensive chemotherapy (IC) is regarded as an effective method to lengthen the OS and to reduce progression risks for these patients (12, 13).

Currently, the National Comprehensive Cancer Network (NCCN) guidelines recommend CCRT in combination with IC for advanced NPC, supported by level IIA evidence (14). However, the application of IC has subsequently become a controversial focus for the treatment of LA-NPC—the main dissent is the increasing toxicity without benefit for the OS of patients (15). As a result, researchers make great efforts to develop reliable tools to address this concern. It is of particular note that, because the proposal of pretreatment EBV-DNA load for the guidance of IC, many studies have explored and uncovered that this biomarker, which sensitively reflects tumor burden, might point out a direction for intensification therapy in LA-NPC (16). This study aimed to investigate the role of pretreatment plasma EBV-DNA load in IC using a meta-analysis, hoping to provide evidence for the use of IC in clinical practice.

## Materials and Methods

### Literature Screening and Search Strategy

All methods were performed according to the Preferred Reporting Items for Systematic Reviews and Meta-Analyses (PRISMA) flow diagram (17, 18). We thoroughly searched all relevant studies updated to May 12, 2020, in PubMed (https://www.ncbi.nlm.nih.gov/pubmed), MEDLINE (https://www.nlm.nih.gov/bsd/medline), Cochrane (https://www.cochranelibrary.com), Embase (https://www.embase.com), and Web of Science (https://www.webofknowledge.com). The search strategy was as follows: ((nasopharyngeal carcinoma) OR (nasopharyngeal cancer) OR (nasopharyngeal neoplasms)) AND ((induction chemotherapy) OR (neo adjuvant chemotherapy) OR (neoadjuvant chemotherapy) OR (new adjuvant chemotherapy) OR (new supplementary chemotherapy) OR (inductive chemotherapy) OR (induced chemotherapy)) AND ((Epstein-Barr Virus DNA) OR (EBV-DNA)). Two authors independently conducted the literature search and initially picked out relevant studies by reading titles and abstracts. Studies describing irrelevant subjects were excluded in the first step. The remaining studies were further screened *via* reading the full texts, and ineligible studies were discarded.

### Inclusion and Exclusion Criteria

Studies could be included if they met the following criteria: (i) subjects were pathologically diagnosed with LA-NPC without distant metastasis or other major complications and received initial treatment; (ii) subjects were assigned to the IC+CCRT and CCRT groups according to pretreatment EBV-DNA load levels, and the efficacy of the two treatment modes was evaluated; (iii) the survival endpoint was OS, progression-free survival (PFS), distant metastasis-free survival (DMFS), or locoregional relapse-free survival (LRFS); (iv) randomized controlled intervention studies and observational studies, such as case-control and cohort studies; (v) all studies were original English-language papers that had already been published with full texts. Studies were excluded if they were reporting (i) literature reviews, case reports, comments, conference proceedings, or animal experiments; (ii) without the data of efficacy between IC+CCRT and CCRT; (iii) without important data (e.g., plasma EBV-DNA loads) for analysis.

### Data Extraction

Two authors independently extracted data from all included studies using standardized forms. Any disagreement was addressed through discussions between them or by a more experienced senior researcher if necessary. The extracted data mainly were clinical characteristics, including publication year, study design, location, sample size, and subjects as well as primary outcomes between IC+CCRT and CCRT, such as the corresponding hazard ratios (HRs) and 95% confidence intervals (CIs) for OS, PFS, DMFS, and LRFS. The two authors directly extracted HRs and 95% CIs from studies or extracted survival rates from survival curves using Engauge Digitizer 6.1 software and calculated HRs and 95% CIs using Tierney’s Excel (19, 20).

### Literature Quality Assessment

Two authors independently assessed the quality of the included studies. Any disagreement was settled through discussion until consensus was reached. Unsettled disagreements were addressed by consultations with the corresponding author. The quality of nonrandomized studies was evaluated using the Newcastle–Ottawa scale (NOS) (21). The quality of randomized controlled trials (RCTs) was assessed using the Cochrane bias assessment tool from the following six dimensions: random allocation, allocation concealment, blinding, research integrity, selective reporting bias, and other bias (22).

### Statistical Analysis

Statistical analysis was performed using Stata 11.0 software (Stata Corporation, College Station, TX), and a *p*-value < 0.05 of summary statistics was considered significant. Heterogeneity was assessed by *I* ^2^ statistics, and the *p*-value of the chi-square test. *I* ^2^ values of 25%, 50%, and 75% were considered as low, moderate, and high heterogeneity, respectively (23). If heterogeneity was not significant (*I* ^2^ *≤* 50% and *p*>0.1), a fixed-effects model was applied; if heterogeneity was significant (*I* ^2^>50% and *p ≤* 0.1), a random-effects model was used to merge the data and assess effect-size indicators. Sensitivity analyses (no less than 3 studies) were repeated by removing one study each time to estimate the effect of a single study on the overall risk. Publication bias was assessed by the funnel plot, and funnel plot asymmetry (no less than 9 studies) was analyzed using Egger’s test.

## Results

### Study Characteristics

**Figure 1:**
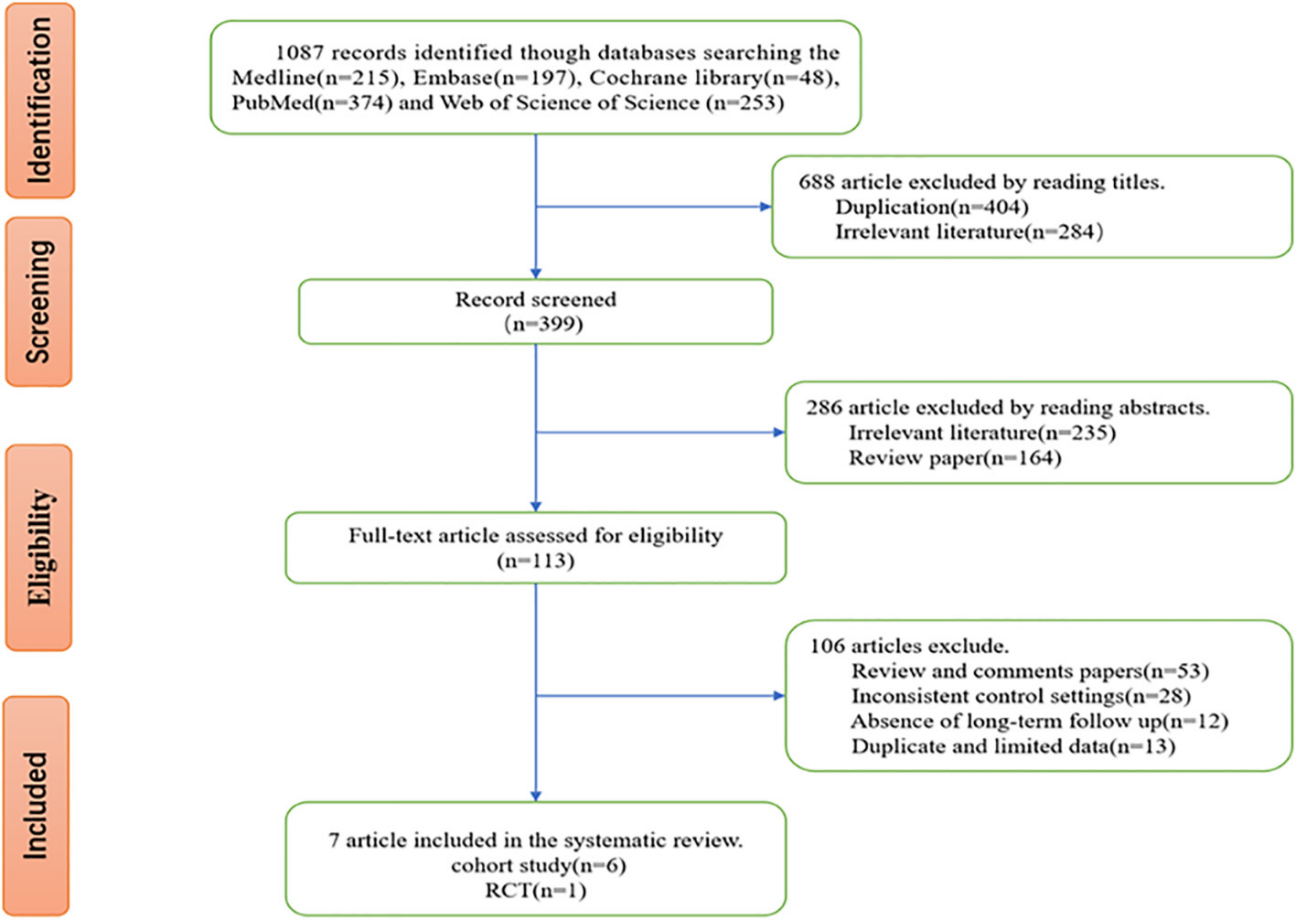
Flow chart of study selection.

After we searched the five databases, we initially acquired 1087 relevant studies, including 215 studies from Medline, 197 studies from Embase, 48 studies from the Cochrane Library, 374 studies from PubMed, and 253 studies from Web of Science. After screening, 7 studies that met the inclusion and exclusion criteria and whose data were extractable were finally included in our meta-analysis. The flowchart of study selection processes is shown in **Figure 1**.

Of the 7 included studies, 6 were observational studies, and 1 was an RCT. All studies were conducted in China with a total sample size of 5289 cases. The minimum sample size of investigation was 121 cases, and the maximum sample size was 1890 cases. Two studies possessed a sample size of more than 1000 cases. All subjects were stage III–IVB NPC (AJCC 7th) or III–IVA NPC (AJCC 8th) patients who received IMRT in combination with cisplatin-based chemotherapy or IC + cisplatin-based CCRT. The pretreatment plasma EBV-DNA load was quantitated using reverse transcription polymerase chain reaction (RT-PCR) analysis in all studies and had distinct cutoff values (0, 1550, 4000, 4650, and 6000 copies/ml). Most studies did not provide detailed illustration of the PCR method. Some studies indicate that the target gene fragment of PCR detection is EBV BamHI-W region. For the convenience of data extraction and merge, the cutoff value at 4000 copies/ml was selected to stratify patients and merge their outcomes. The median time of follow-up varied from 38.7 to 71.5 months (**Table 1**).

**Table 1:** Characteristics of All Included Studies.

Study ID	Country	Study Design	Sample Case						Radiation	IC	TNM(AJCC)	Follow-up(months)	Outcome
			Total	Gender(M/F)	EBV-DNA≥4000 copies/ml		EBV-DNA<4000 copies/ml						
					IC+CCRT	CCRT	IC+CCRT	CCRT					
Du et al. (24)	China	Retrospective	881	674/207	284	152	186	259	IMRT	platinum-basedregimen	III–IVB(7th)	38.7	PFS, DMFS
Peng et al. (25)	China	Retrospective	290	217/73	NA	NA	145	145	IMRT	PF, TP, TPF	III-IVB(7th)	NA	OS, DFS, DMFS, LRFS
Guo et al. (26)	China	Retrospective	156	118/38	79	77	NA	NA	IMRT	PF	III-IVB(7th)	51.3	OS, PFS, DMFS, LRFS
Jin et al. (27)	China	Retrospective	639	482/157	NA	NA	296	343	IMRT	PF	II-IVB(7th)	58	OS, PFS, DMFS, LRFS
Zhang et al. (28)	China	Retrospective	1890	1364/526	945	945	NA	NA	IMRT	PF, TP, TPF	III-IVA(8th)	NA	OS, DFS, DMFS, LRFS
Liu et al. (29)	China	Retrospective	1312	NA	369	205	303	435	IMRT	PF, TP, TPF	III-IVB(7th)	NA	OS, PFS, DMFS
Li et al. (30)	China	RCT	121	NA	58	63	NA	NA	IMRT	TPF	III-IVB(7th)	71.5	OS, FFS

NA, Not applicable; EBV-DNA, Epstein-Barr virus deoxyribonucleic acid; IC, induction chemotherapy; CCRT, concurrent chemoradiotherapy; IMRT, intensity modulated radiation therapy; PF, cisplatin and fluorouracil; TP, docetaxel and cisplatin; TPF, docetaxel, cisplatin and fluorouracil; OS, overall survival; PFS, progression free survival; DMFS, distance metastasis free survival; LRFS, local recurrence free survival; DFS, disease free survival; FFS, failure free survival; TNM (AJCC), American Joint Committee on Cancer Tumor-Node-Metastasis Staging for Nasopharyngeal Cancer (7th/8th edition).

### Quality Assessment

Of all included studies, 6 observational studies received NOS scores of ≥6, and one RCT was at moderate risk of bias. Generally, most studies were high quality in our analysis (**Table 2**).

**Table 2:** The Risk of Bias and Quality of All Included Studies.

Study	Selection				Comparability	Outcome			NOS
	Representativeness of the exposed cohort	Selection of the nonexposed cohort	Ascertainmentof exposure	Demonstration that outcome of interest was not present at start of study	Comparability of cohort based on the design or analysis	Assessmentof outcome	Was follow-up long enough for outcome to occur	Adequate offollow up ofcohort	
Du et al. (24)	1	1	1	0	0	1	1	1	6
Peng et al. (25)	1	1	1	0	2	0	1	1	7
Guo et al. (26)	1	1	1	0	2	0	1	1	7
Jin et al. (27)	1	1	1	1	0	1	1	1	7
Zhang et al. (28)	1	1	1	0	2	0	1	1	7
Liu et al. (29)	1	1	1	0	2	0	1	1	7
**Study**	**Random sequence generation**		**Allocation concealment**		**Blinding of participants and personnel**	**Incomplete outcome data**		**Selective reporting**	**Other bias**
Li et al. (30)	+		+		?	?		+	+

### Meta-Analysis and Heterogeneity Test

A cutoff value of 4000 copies/ml of pretreatment EBV-DNA was used to define a high (≥4000 copies/ml) or low (<4000 copies/ml) load. The OS, PFS, DMFS, and LRFS of patients after IC+CCRT or CCRT treatment were separately merged for the heterogeneity test.

**Figure 2:**
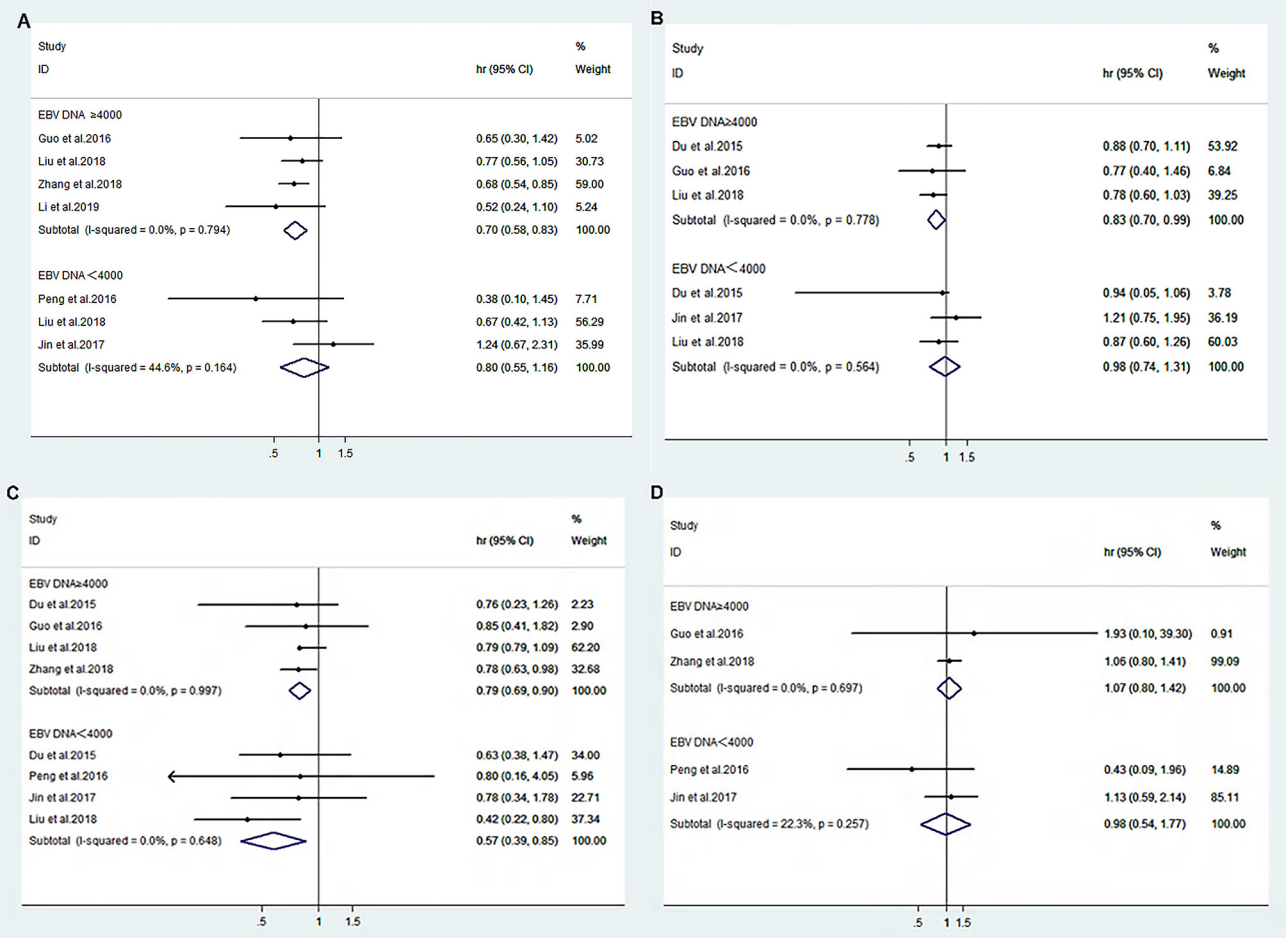
Forest plot of overall survival **(A)**, progression-free survival **(B)**, distance metastasis-free survival **(C)**, and locoregional recurrence-free survival **(D)** between IC+CCRT and CCRT based on EBV-DNA ≥4000 copies/ml or EBV-DNA <4000 copies/ml.

The heterogeneity test for the efficacy outcome revealed the homogeneity of OS (*I* ^2 ^= 0.0%, *p*=0.794), PFS (*I* ^2 ^= 0.0%, *p*=0.778), DMFS (*I* ^2 ^= 0.0%, *p*=0.997), and LRFS (*I* ^2 ^= 0.0%, *p*=0.697) in patients with pretreatment EBV-DNA loads of ≥4000 copies/ml in both the IC+CCRT and CCRT groups (**Figures 2A–D**). A fixed-effects model was used for the meta-analysis of the efficacy outcome. The results show that IC+CCRT significantly extended the OS (HR 0.70 [95% CI 0.58-0.83], *p*=0.000), PFS (HR 0.83 [95% CI 0.70-0.99], *p*=0.033), and DMFS (HR 0.79 [95% CI 0.69-0.9], *p*=0.000) of patients compared with the CCRT alone, but there were no benefits to LRFS (HR 1.07 [95% CI 0.80-1.42], *p*=0.647) (**Table 3**).

**Table 3:** Summary of Meta-Analysis Results of IC+CCRT versus CCRT.

EBV-DNA Cutoff	Outcomes	No. of studies	Model	HR (95% CI)	*p*	Heterogeneity (*I* ^2^, *p*)
≥4000 copies/ml	OS	4	Fixed	0.70 (0.58-0.83)	0.000	0.0%,0.794
	PFS	3	Fixed	0.83 (0.70-0.99)	0.033	0.0%, 0.778
	DMFS	4	Fixed	0.79 (0.69-0.90)	0.000	0.0%, 0.997
	LRFS	2	Fixed	1.07 (0.80-1.42)	0.647	0.0%, 0.697
<4000 copies/ml	OS	3	Fixed	0.88(0.55-1.16)	0.240	44.6%, 0.164
	PFS	3	Fixed	0.98 (0.74-1.31)	0.908	0.0%, 0.564
	DMFS	4	Fixed	0.57 (0.39-0.85)	0.006	0.0%, 0.648
	LRFS	2	Fixed	0.98 (0.54-1.77)	0.943	22.3%, 0.257

EBV-DNA, Epstein-Barr virus deoxyribonucleic acid; HR, hazard ratio; CI, confidence interval.

The heterogeneity test for the efficacy displayed nonsignificant heterogeneity of OS (*I* ^2 ^= 44.6%, *p*=0.164), PFS (*I* ^2 ^= 0.0%, *p*=0.564), DMFS (*I* ^2 ^= 0.0%, *p*=0.648), and LRFS (*I* ^2 ^= 22.3%, *p*=0.257) in patients with pretreatment EBV-DNA loads of <4000 copies/ml (**Figures 2A–D**). Therefore, a fixed-effects model was selected for the meta-analysis, and the results suggest that IC+CCRT prolonged DMFS (HR 0.57 [95% CI 0.39-0.85], *p*=0.006) without significant improvements in OS (HR 0.88 [95% CI 0.55-1.26], *p*=0.240), PFS (HR 0.98 [95% CI 0.74-1.31], *p*=0.908), and LRFS (HR 0.98 [95% CI 0.54-1.77], *p*=0.943) of patients compared with CCRT (**Table 3**).

### Sensitivity Analysis and Publication Bias

**Figure 3:**
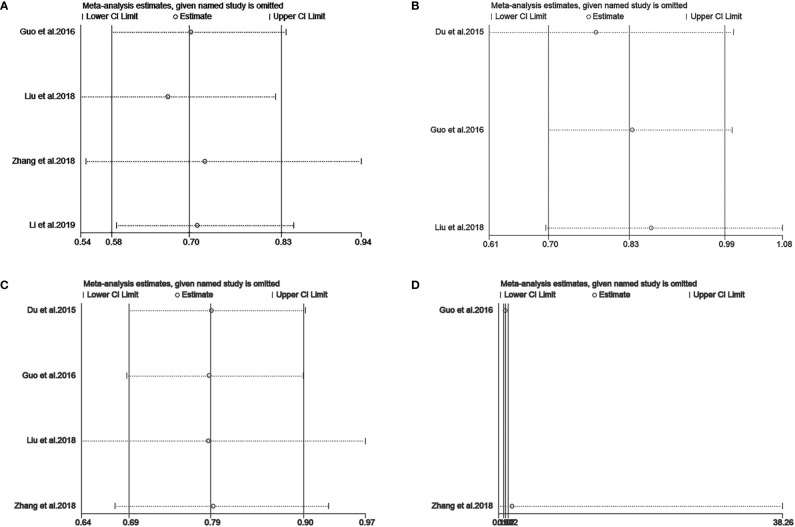
Sensitivity analysis of overall survival **(A)**, progression-free survival **(B)**, distance metastasis-free survival **(C)**, and locoregional recurrence-free survival **(D)** between IC+CCRT and CCRT based on EBV-DNA ≥4000 copies/ml.

**Figure 4:**
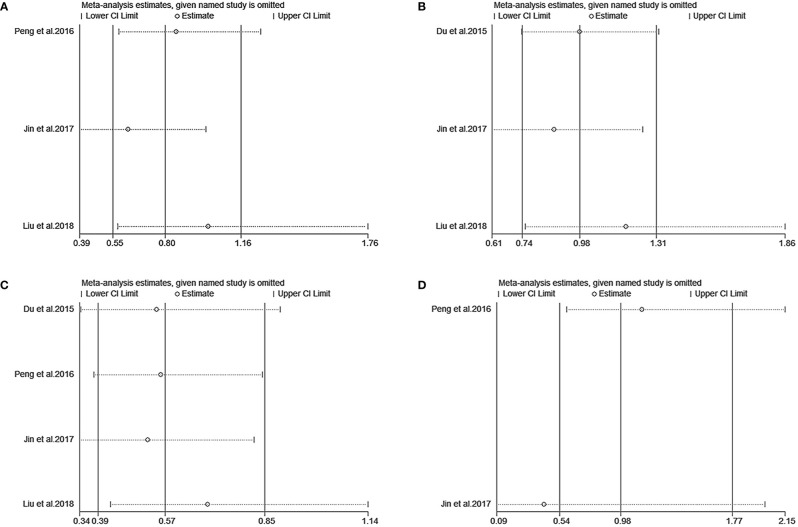
Sensitivity analysis of overall survival **(A)**, progression-free survival **(B)**, distance metastasis-free survival **(C)**, and locoregional recurrence-free survival **(D)** between IC+CCRT and CCRT based on EBV-DNA <4000 copies/ml.

We performed a sensitivity analysis to measure the stability and reliability of the data. The results demonstrate that IC+CCRT had more stable efficacy than CCRT in OS, PFS, DMFS, and LRFS of patients with pretreatment plasma EBV-DNA ≥4000 copies/ml (**Figures 3A–D**). In addition, IC+CCRT exhibited more reliable efficacy than CCRT in OS, PFS, DMFS, and LRFS of patients with pretreatment plasma EBV-DNA <4000 copies/ml (**Figures 4A–D**). The assessment of publication bias was unavailable as the number of included studies for each meta-analysis was less than 9.

## Discussion

This study contains LA-NPC patients classified as stage III–IVB NPC by AJCC 7th or stage III–IVA NPC by AJCC 8th and investigates the clinical value of the emerging biomarker pretreatment EBV-DNA load in the use of IC. EBV-DNA was detected by PCR methodologically in all of the included studies. We, for the first time, probe the role of pretreatment plasma EBV-DNA load as a guide for IC in LA-NPC through meta-analysis, and the results show that high-risk patients who had a pretreatment EBV-DNA load ≥4000 copies/ml were more likely to benefit from IC. However, low-risk patients who had a pretreatment EBV-DNA load <4000 copies/ml might not benefit from IC.

With the great improvement of focal therapy for cancer control in the era of IMRT, distant metastasis has become the major cause of treatment failure. Thus, studies on the long-term efficacy of NPC treatment have gradually turned to systemic drug therapy from focal therapy (31), and the addition of IC to the basic treatment of CCRT for LA-NPC has been applied in clinical practice (32, 33). However, it is very urgent to stratify the high-risk populations as the OS of all patients cannot be significantly ameliorated after IC (34). The prognostic value of the pretreatment EBV-DNA load in NPC has been confirmed by many researchers. The majority of studies show that high pretreatment EBV-DNA load is a predictor of poor OS, PFS, or DMFS and a risk factor for distant metastasis compared to low pretreatment EBV-DNA load (35, 36). Currently, anti-NPC regimens are adopted according to the sites of tumor invasion and TNM staging. However, tumor heterogeneity also affects the efficacy among patients at this stage (37). In addition, there are a number of studies on the prognostic value of plasma EBV-DNA levels in NPC. In 2000, researchers in Hong Kong confirmed that plasma EBV-DNA load was an independent predictor of metastasis within 1 year after treatment for NPC, and the load level was closely associated with the risk of cancer death; a 10-fold increase in the load level brought about a 1.6-fold increase in the risk of death (5). In 2004, Lin et al. found that the 2-year OS (100% vs. 88.8%) and RFS (83.4% vs. 66.4%) rates in patients who had a pretreatment EBV-DNA load <1500 copies/ml were higher than those in patients who had a pretreatment EBV-DNA load ≥1500 copies/ml (38). All of these findings provide a novel strategy to guide individualized treatment by using pretreatment EBV-DNA load.

In this meta-analysis, the combination of IC and CCRT offered longer PFS and OS for LA-NPC patients who had a pretreatment plasma EBV-DNA load ≥4000 copies/ml without heterogeneity of the efficacy outcome. Sensitivity analysis showed the stable efficacy of IC+CCRT. These results confirm that IC followed by CCRT is an indispensable strategy for patients with a high EBV-DNA load. For patients who had a pretreatment EBV-DNA load <4000 copies/ml, IC did not provide better OS or PFS. Although there was heterogeneity of merged effect sizes in terms of OS, the heterogeneity was nonsignificant (*I* ^2^ <50%), and efficacy was stable after sensitivity analysis. These results suggest that IC+CCRT cannot improve the PFS and OS of patients with a low EBV-DNA load. Perhaps other combination treatments based on CCRT might be more effective for these low-risk patients. In addition, our findings may aid in guiding medical strategies in clinical practice. Overall, regardless of a pretreatment EBV-DNA load ≥4000 or <4000 copies/ml, the combination of IC and CCRT contributes to improved DMFS but not LRFS compared with CCRT alone. The reason may account for intensive chemotherapy, which mainly plays a role for distant metastasis and has less impact on the risk of local recurrence. This finding highlights the importance of IC for the control of distant metastasis and the optimization of the control of localized or regional lesions. Of note, the improvement of DMFS and LRFS after IC+CCRT has no relation to pretreatment plasma EBV-DNA loads. This outcome indicates that there is great room for investigating more sensitive and reliable prognostic markers based on pretreatment plasma EBV-DNA load.

Recently, more researchers have begun to explore the value of IC in LA-NPC on the basis of pretreatment EBV-DNA load. Du et al. (24) first recognized that pretreatment plasma EBV-DNA load could aid in decision making rather than TNM stage to guide the selection of IC. The authors believed that IC prolonged the DMFS and PFS of N2-3 stage NPC patients with an EBV-DNA load ≥4000 copies/ml. These patients were defined as a very high-risk population who benefited from introduction IC by Guo et al. (26). Subsequently, Peng et al. (25) defined patients with a pretreatment plasma EBV-DNA load <1500 copies/ml as low-risk populations and believed that the introduction of IC did not improve OS, DMFS, PFS, or LRFS of these patients. Similarly, Jin et al. (27) also selected low-risk patients with a pretreatment plasma EBV-DNA load of 0 copies/ml for analysis and yielded consistent results. Zhang et al. (28) first stratified LA-NPC patients by cutting off pretreatment plasma EBV-DNA. The results show that low-risk patients (EBV-DNA <4650 copies/ml) receiving IC obtained no amelioration in OS, DMFS, or LRFS, and high-risk patients (EBV-DNA ≥4650 copies/ml) could benefit from IC in terms of OS and DMFS. Regarding whether patients with a high EBV-DNA load may benefit from IC, a phase III RCT of the optimum IC shows that the OS in the subgroup of patients whose pretreatment plasma EBV-DNA >6000 copies/ml could be significantly improved after IC with docetaxel, cisplatin, and fluorouracil (TPF) regimens (30). However, a study reports that IC promoted DMFS but not OS or PFS in N0-1 of stage III–IVB (AJCC 7th) NPC patients with an EBV-DNA load <4000 copies/mL (the low-risk group). N2-3 patients who had EBV-DNA ≥4000 copies/ml (the high-risk group) did not benefit from IC in terms of OS, PFS, and DMFS (29). Although the cutoff value of pretreatment plasma EBV-DNA was distinct among the above studies, they highlight that pretreatment plasma EBV-DNA could serve as an effective marker in the stratification of a population suitable for IC.

For the first time, we conducted a meta-analysis of plasma EBV-DNA stratifying LA-NPC patients into high- (a high plasma EBV-DNA load) and low-risk (a low plasma EBV-DNA load) groups and compared the efficacy of IC+CCRT vs. CCRT alone in these patients. Our study provided evidence that plasma EBV-DNA had great potential in guiding the use of IC. However, there are some limitations in our study. First, the most included studies are case-control studies, and more prospective randomized controlled studies will be included to make our conclusion more reliable. Second, selection bias is inevitable in the literature-screening processes because unpublished and ongoing publication studies could not be included. Third, studies on the relevant topic are limited, and there is great room for further exploration in this area.

## Conclusion

Pretreatment plasma EBV-DNA load can serve as a promising effective marker to guide the selection of IC based on current CCRT strategies for LA-NPC patients. In our study, IC could prolong OS and PFS among patients with a pretreatment plasma EBV-DNA load ≥4000 copies/ml but not in patients with a pretreatment plasma EBV-DNA load <4000 copies/ml. Moreover, our results demonstrate that, regardless of the pretreatment plasma EBV-DNA load, IC could improve DMFS and provides no benefit for LRFS, meaning that it may decrease the risk of distant metastasis and have no effect on reducing local or regional relapse. Hence, it is worth conducting prospective studies to explore and verify these findings further.

## Data Availability Statement

The original contributions presented in the study are included in the article/supplementary material. Further inquiries can be directed to the corresponding author.

## Author Contributions

XZ: study conception and design. LL, XYC, CZ, and LC: literature screening and quality assessment. LL, XYC, XSC, and GT: data extraction and data analysis. LL and XYC: manuscript writing and manuscript revision. All authors contributed to the article and approved the submitted version.

## Funding

This research was supported by National Natural Science Foundation of China (81760544), Key Research and Development Program Project of Guangxi Zhuang Autonomous Region (Grant No. GuikeAB18221007), Independent Project of Key Laboratory of Early Prevention & Treatment for Regional High‐Incidence‐Tumor (Grant No. GKE2019‐17), Self-Raised Scientific Research Fund of the Ministry of Health of Guangxi Province (No. z2015434), and Science Foundation for Distinguished Young Scholars of Guangxi University of Chinese Medicine (No. 2020JQ001).

## Conflict of Interest

The authors declare that the research was conducted in the absence of any commercial or financial relationships that could be construed as a potential conflict of interest.
